# Supplementary material for: Scaffold-Lab: Critical evaluation and ranking of protein backbone generation methods in a unified framework
Source: PLoS Comput Biol. 2026 May 22;22(5):e1014290. doi: 10.1371/journal.pcbi.1014290 (PMC13225634; doi:10.1371/journal.pcbi.1014290)
Supplement: S1 File — S1 Fig. Compared studies on different sequence design methods. To make a comparison between ProteinMPNN and ESM-IF1, we randomly selected 20 backbones generated for each tested length (from 50 to 1000) and designed 10 sequences for each backbone, resulting in a subset containing 9 (number of unique lengths) × 20 = 180 unique backbones and 180 × 10 = 1800 designed sequences. We then feed these sequences into ESMFold and calculated the values of self-consistency TM-score (sc-TM). (A) Correlation between ProteinMPNN and ESM-IF1. Each point in the plot denotes a unique backbone, x and y axis denote the sc-TM values origined from ProteinMPNN and ESM-IF1, respectively. The degree of color denotes the number of residues of each sequence. The value displayed on the lower right denotes the Spearman correlation between results from different sequence design methods. (B) Distribution of sc-TM values from ProteinMPNN and ESM-IF1 are displayed in a boxenplot. It can be observed that sequences obtained from ProteinMPNN exist significant higher sc-TM values than ESM-IF1 no matter from the perspective of single backbones or overall distribution. S2 Fig. Correlation between three protein structure prediction methods. For comparison, we randomly selected sequences designed by ProteinMPNN from generated backbones and fed them into three structure predictors. For each length of unconditional sampled backbones, we randomly selected 100 sequences, which resulted in a total number of 900 sequences predicted by ESMFold, AlphaFold2 (without MSA) and OmegaFold, respectively. Each point in the subplot denotes a unique sequence and the value displayed on the lower right denotes the Spearman correlation between results from different structure predictors. The degree of color denotes the number of residues of each sequence. (A) Correlation between ESMFold and AlphaFold2 (without MSA). (B) Correlation between AlphaFold2 (no MSA) and OmegaFold. (C) Correlation between ESMFold and OmegaFold. S3 Fig [file pcbi.1014290.s001.docx]

**Response letter for**

**Scaffold-Lab: Critical Evaluation and Ranking of Protein Backbone Generation Methods in A Unified Framework**

March 5, 2026

**Manuscript Number:** PCOMPBIOL-D-25-02477

Dear Editors:

We would like to thank the editors and reviewers for their critical assessments and constructive feedback and suggestions upon our manuscript. These comments provide significant viewpoints and help us improve the quality of our manuscript under its current shape, both in terms of the solidity of our experiments, the depth of discussion and the language flow throughout the manuscript.

We have carefully gone through each single point of the items brought by the editors and reviewers. We want to first summarize our modifications in this revised version below:

1. **Missing contents:** We have added the missing content raised by Reviewer #1 back in the right place of Supplementary Information.
2. **MolProbity analysis:** We have conducted the structural validity assessments using MolProbity upon the generated backbones from tested methods, which was raised by Reviewer #3, with additional analysis and discussions both in the main text and Supplementary Information.
3. **Further exploration in conditional design:** Based on the comments of Reviewer #3, we have conducted an active-site conditioned design using the four methods tested in conditional generation with the active site of an artificial retro-aldolase, RA95.5-8F, to test the one-shot performances of different methods. We have also included the discussion and analysis related to this version both in main text and Supplementary Information.
4. **Better distinctions between different scenarios of loop contents:** We have now used more proper descriptions upon the loop contents described in our manuscript both in the part of Structural Properties and in Discussion based on the advice from Reviewer #3.
5. **Corrected and additional figures:** We have corrected the annotation of significant test and have added a plot depicting the ranking values of different methods based on the comments from Reviewer #3.
6. **Justification of language:** We have now adjusted the language in the initial manuscript to make it clearer expressed and presented.

We have provided the following files in the revision following the guidelines of PLOS Computational Biology:

1. A “**Response to Reviewers**” (This file) highlighting the changes we’ve made in the updated manuscript, and the
2. A “**Manuscript**” file containing an updated version of the manuscript.
3. A “**Revised** **Manuscript with Track Changes**” file highlighting the edits we’ve made during revision.
4. A “**Supplementary Information**” file which is an updated version of Supplementary Information.

Next, we are going to respond to the comments from the reviewers in a point-by-point manner. The original comments from the Reviewers are in Times New Roman and the our replies are in Arial.

Reviewer #1: On pg. 4 of SI it appears that some text might be missing. The authors write:

"We additionally adapted two modifications: "

after which the text moves onto a new paragraph.

**Author Replies:** We would like to thank the reviewer for the detailed pass of our manuscript and pointing this out. We have indeed missed some context here. The lacked content is here:

*“... We additionally adapted two modifications:*

1. *We used sc-TM instead of sc-RMSD throughout our evaluation pipeline. Although many works used a threshold of sc-RMSD < 2.0 Å to define the success of backbones, we found this metric showcased commonly high values and hard for scaling during preliminary experiments, while sc-TM showed a greater robustness. Nevertheless, we calculated the designability and success rate using sc-RMSD for a comparison and the integrity of our evaluation, which can be found in Supplementary Figure 3.*
2. *“Top N strategy”. While current works consider a backbone as success when there is solely one sequence that satisfies the criteria, we argue that the number of sequences that can fold into the generated structures reveal the robustness and fitness of backbones to some extent. So during our experiment, we reported the sc-TM as the mean value of 3 sequences with highest sc-TM among generated sequences, where the formula is defined as follows:*

$$Success_{backbone}=\left\{ \begin{aligned} 1,\frac{1}{N}\sum_{i=1}^{N} scTM>0.5 | (scTM_{1}>\ldots>scTM_{N}>scTM_{i}) \\ 0, others \end{aligned} \right.$$

*During our evaluation, we chose N=3, which means we calculated the designability using the mean value of top 3 sequences with highest sc-TM values out of 10 selected sequences. We also reported the distribution of sc-TM and success rate by picking the best sequence as a fair comparison, which are displayed in Figure 2B and Supplementary Figure 5-6.”*

We have now added them back in the corresponding part in **Page 4-5 of Supplementary Information.** We would like to thank the reviewer again for the detailed look of catching this missing contents.

Reviewer #2: In this manuscript, “Scaffold-Lab: Critical Evaluation and Ranking of Protein Backbone Generation Methods in A Unified Framework,” Authored by Zheng et al., the authors comprehensively evaluated protein backbone generation methods across various protein sizes and scaffolds and provided a systematic framework for evaluating Protein design methods. They have used extensive properties to rate and rank the available methods for protein design into multiple categories. They found that RFdiffusion performed most consistently across proteins of varying lengths. However, based on their evaluation metrics, the authors have concluded that none of the discussed methods is well-suited to all categories. Overall, these methods are biased towards generating more structured proteins and have limited capabilities to design loops and intrinsically disordered regions. They suggest users must design a framework tailored to the length and biological function of their protein sequence.

An interesting point to note is that, according to the evaluation in this paper, the authors conclude that novelty in a designed protein structure comes at the expense of its designability, questioning the emphasis on novelty in protein backbone generation methods. In general, low designability indicates that the structure is farther from the existing protein, and hence, the feasibility of in vivo expression of such proteins might be problematic. Hence, the authors have questioned the overemphasis on the novelty of designed proteins and suggest that designability of the motif scaffold is a prerequisite when users are interested in a certain biological function.

**Author Replies:** We sincerely thank the reviewer for the positive feedback of our manuscript and the thorough summary provided. Specifically, the discussion related to the trade-off of novelty and designability is important; we believe designing novel protein structures do have merits in exploring novel folds in protein structural space, and thus potentially novel functions. Nevertheless, overemphasis on the novelty over designability might not be the best choice since it might risk lower designability. One realistic challenge is that protein backbone generative methods typically need fixed-backbone sequence design methods and structure prediction methods to participate in the computational design oracle. Since these methods are typically trained with existing proteins, this might also hinder the evaluation designability. Consequently, we suggest users to explicitly take designability into consideration when comparing the novelty of different methods. Thank you again for mentioning this specific point during the review process.

Reviewer #3 (Academic Editor)

Zheng et al describe Scaffold-Lab, a unified framework for systematic evaluation of protein  backbone generation methods. They evaluated several representative methods and provide a detailed analysis of their performance and utilities. Overall, RFdiffusion performed the best among all tested methods. Other methods also demonstrated reliable performance on short (< 300 residues) proteins

Considering the rapid advances in AI methods for de novo protein design, the authors are correct to point out there is a  need for standardized benchmarks to assess protein backbone generation methods. Scaffold-Lab provides unified framework for the evaluation of protein backbone generation methods. The results clearly demonstrate the challenge for most methods is "designability" for targets > 300 residues, and the unique strengths and weaknesses of RFDiffusion. The criteria for assessing various methods and protocols are also useful. Although the study is not as comprehensive as one might like, it does provide a significant and valuable effort in this direction.

**Author Replies:** We would like to express our appreciation for the positive feedback from the reviewer. We especially want to thank the reviewer for the constructive suggestions in improving the manuscript over its current shape, which prompted us to bring additional analysis, discussions and a revised manuscript upon the previous version.

A revised manuscript should address the following issues.

1. The authors state in the final paragraph of the Introduction: "We observed that the ability to create an ideal scaffold is a prerequisite of successful designs than simply meeting certain conditional criteria" The language here is not clear. Since this is a key point - it should be made more clearly.

**Author Replies:** We would like to thank the reviewer(s) for this suggestion. We have now rephrased it into a clearer version to emphasize that the task of motif reconstruction is a relatively more difficult task than the task of generating a designable scaffold: “*We observed the task of motif reconstruction is a more difficult task than the task of generating a designable scaffold, and that creating a successful overall scaffold is a prerequisite for effective motif handling.*” We hope that it is clearer now.

2. For "structural properties", since the proteins are refolded with ESM_Fold or AlphaFold, could the authors also consider an empirical packing score like Molprobity? This has the advantage also that it is quantifiable.

**Author Replies:** Thank you for this valuable suggestion. We believe an empirical packing score like MolProbity could evaluate the structural validity of designed backbones. Although we should claim that the analysis upon the “Structural Properties” section was directly performed on backbones but not refolded structures, since the goal is to explore the behaviour of backbone generated methods without the bias inherited from protein structure prediction methods. Nevertheless, we believe the structural validity is an additional point to be explored, so we have used Molprobity to check the structural validity of generated backbones.

We installed MolProbity from its GitHub repository (<https://github.com/rlabduke/MolProbity>) locally and used the one-line-analysis command to examine the structural validity (please refer to <https://github.com/rlabduke/MolProbity/blob/master/doc/command_line_tools.txt> for more details) of the same set of protein backbones we tested in the manuscript. We excluded Genie as the same to the analysis of structural properties since its designed backbones only contain carbon-alpha atoms. For the rest of the four methods, only Chroma possesses the designed sequences and sidechains, while RFdiffusion, FrameDiff and FrameFlow only contain backbone atoms, so the statistics of rotamers could not be calculated for them. Thus, we calculate the following metrics to explore the structural validities of different methods using MolProbity:

1. **Clash score:** This is defined by MolProbity as the number of serious steric overlaps (> 0.4 Å) per 1000 atoms within an analyzed structure, and used to quantify the plausibility of atomistic overlaps. A lower clash score typically indicates the tested protein contains less unflavoured clashes, thereby more acceptable steric contacts. Though there is not an absolute cutoff of an acceptable clash score, it could be inferred from the Figure 6 of the MolProbity paper in 2018 [1] (see below) that high-quality reference crystal structures typically obtained a clash score less than 25.

From the figure below we can see that the clash scores exhibit significant differences between different methods, and showcased an increasing trend from shorter to longer proteins. RFdiffusion and FrameFlow have most of their backbones lower than the 25.0 cutoff from the Top8000 high-quality reference dataset, with RFdiffusion having substantially low clash scores.

We note that the high clash scores of Chroma might stem from that it produces all-atom protein backbones during the design process, where the clashes in sidechains may contribute significantly to the overall clash scores. Apart from Chroma, we hypothesize that the exceptional performance of RFdiffusion in clash score could be owed to the fact that it was fine-tuned from RoseTTAFold, whose training loss contained a van der Waal loss penalizing the clashes.

**Figure 1. Clash scores of different methods.** The vertical dashed lines indicate the mean clash score for backbones within each method.

2. **Geometric rationality of covalent bond lengths and covalent bond angles:** MolProbity calculated the outliers within each evaluated protein, where outliers are defined as those differ from the expected values by more than 4𝞂. The goal of an idealized structure is to have zero bad bonds and bad angles less than 0.1%, respectively. Here we calculated the percentage of bad bonds and bad angles, which are displayed below. We can see that most backbones designed by current methods obtained bad angles and bad bonds beyond the expected acceptable thresholds, suggesting that the modeling techniques of the covalent geometry are yet to be improved. Chroma is the only one consistently having a distribution close to zero, in contrast to its performance in clash score. This also suggests that different methods have unique properties towards different aspects of geometric properties, probably due to different training schemes of them.

**Figure 2. Percentage of bad bonds for different methods.** The vertical dashed lines indicate the mean values for the backbones of each method.

**Figure 3. Percentage of bad angles for different methods.** The vertical dashed lines indicate the mean values for the backbones of each method.

In general, we concluded that while most of the methods are capable of generating idealized protein structures, they have yet to be improved in structural validity in terms of geometric details. We also discovered that different methods have their specialized aspects of geometric details, for example, RFdiffusion for modeling idealized atom-atom contacts and Chroma for modeling idealized bond lengths and angles, probably due to different training strategies. This provides insights of modeling idealized geometric details of designed structures for future methods.

Finally, we plan to add this part of analysis on top of the current form of the “Structural Properties” section, with the following text accompanied with figures described previously in **Page 17**:

*“****Structural validity.*** *While most of the methods are capable of generating protein backbones with idealized secondary structures and idealized globular shape, the local geometric details of inter-residue contacts and rationality of biophysical properties are yet to be explored. Thus, we examined the structural validities of tested methods with MolProbity [1] in terms of clash score and the percentage of bad angles and lengths. It could be observed from Figure 1 that the clash scores of different methods increased over the length of generated backbones and the overall distribution is higher than the idealized values of high-quality referenced crystal structures [1], with the exception of RFdiffusion obtaining a significantly lower clash score than different methods. However, in terms of bad bonds and bad angles, Chroma performed the best among all tested methods with the distribution close to zero, with the others having a percentage ranging from 0% to 20%, far beyond the expected threshold of 0% for bad bonds and 0.1% for bad angles, respectively. (Figure 2 and 3).*

*We hypothesize the differences between methods in terms of various aspects of structural validity may be attributed to the differing training schemes of the methods tested, as well as the different representation schemes of generated backbones. For instance, Chroma has an internal sequence design module which is capable of generating backbones with sidechain atoms, which might contributed to its high clash scores. Additionally, this brings out the fact that beyond the idealized overall protein structures, the local biophysical properties of current generative models still have room for improvements. This could be achieved by introducing auxiliary losses specialized for optimizing geometric details locally for both inter-residue and intra-residue contacts.”*

* Note: The number of referred figures and references are the ones in the response letter; those are changed in the revised manuscript in accordance with the overall numbering scheme.

3. In Methods the author state "For each unique length within each method, we generated 100 backbones with 10 sequences for each backbone to be evaluated through a refolding pipeline." This seems to relate to the Results of Fig 2 and 3, but not for the motif-scaffolding study which uses the 24-case benchmark of RFdiffusion. Perhaps this point is better made in the legends of Fig 2 and 3

**Author Replies:** Thank you for pointing this out. We have now adjusted the text in the “Materials and Methods” section and added a paragraph to better describe different evaluation schemes for unconditional generation and motif-scaffolding, respectively. We have also added the text in the captions of **Figure 2 and 3** in the main text to make the points clearer.

4. Panel 2F. The only stars shown are ***. It appears contradictory that the distributions for Medium RFDiffusion with and without penalty look nearly the same, but have p < 0.001, while for Medium FrameDiff the distributions look different but are not statistically different. Is this all correct?

We would like to thank the reviewer for pointing this out. Indeed, the stars in the “Medium” group were mistakenly annotated, where the methods with significantly different scores are FrameDiff and Chroma. We have corrected this and revised the figure, and the data of significance test is also presented in **Supplementary Table 2**.

**Figure 4. Corrected Figure 2E displaying novelty scores w/ and w/o penalties from designability.**

5. Both the Analysis of Structural Properties and the last paragraph of Discussion do not properly distinguish "ordered non-regular structures" from "intrinsically-disordered" regions. These are treated as synonymous, which they are not. Are the designed regions designated as "coil" non-regular ordered regions or disorder regions?

**Author Replies:** Thank you for this insightful question. We assigned the secondary structures with DSSP, and we agree that ordered non-regular structures and intrinsically-disordered regions are different in aspects of structural contents and should be distinguished from each other. In fact, we did intended to separate different contents of coil / loops from each other; for instance, in the analysis regards to secondary structures, we make “turns” (annotated by DSSP as “T”) as a standalone type of secondary structure contents, for they typically being connections between ordered secondary structures and are akin to the “coil” non-regular ordered regions. As for the remaining content of loops, since we performed the secondary structure analysis upon each of the single static backbones using DSSP, it is non-trivial for us to know whether the loop contents are relatively stable or intrinsically disordered over a certain time scale without experimental determination or molecular dynamics simulation. Since loop contents are generally the most flexible regions within a protein and could serve as difficult functional roles such as surface-exposed loops for catalytic interaction as well as intrinsically ordered regions for binding other molecules [2], we argue that designing protein backbones with those flexible regions could be of great potentials of protein engineering.

Thus, we decide to rephrase the context we used in the previous version.

In “Structural Properties”:

**Page 17** *“...in contrast to the long-range found in β-sheets and the flexible interactions in coils.” -> “...in contrast to the long-range interactions found in β-sheets and more irregular regions like loop contents.”*

**Page 17** *“...this approach may limit the modeling of flexible loop regions and intrinsically ordered proteins (IDPs), which are essential for regulating protein functions and enzyme catalytic activities.” -> “...this approach may limit the modeling of more irregular regions like ordered loops and intrinsically ordered regions, both essential for regulating protein functions and enzyme catalytic activities.”*

In “Discussion”:

**Page 23** *“... with limited capability to generate coil regions or IDPs -> with limited capability to generate loop contents, which could typically be flexible regions in proteins and have potential to interact with other biomolecules [2].”*

We hope the refined version of text could better distinguish different categories of loops contents.

6. Ranking. It would be nice to have a histogram plot for Mean Ci vs method, ordered by decreasing Ci score. This would make it clear which methods were best scoring. One histogram plots could combine the data for short, medium, and long lengths.

**Author Replies:** Thank you for your suggestions. We have now added a plot depicting the mean C_i vs method in a descending order for each group in the **Figure 5** displayed below, and added to the refined version as **Figure 4 in the main text**. We hope this additional plot would make the ranking of different methods more illustrative.

**Figure 5. Mean TOPSIS score (C_i, the calculated relative nearness value of TOPSIS results) of different methods.** The numbers upon each bar denotes the mean value across different lengths within the length group.

7. The observed correlation between successful non-conditional designability and successful conditional motif scaffolding is very important, though perhaps obvious to some. The paper would be stronger if more additional assessments were made of different approaches to conditional design, and/or applications to specific cases of binder or active site design.  It seems that exploring strengths and weaknesses of different methods and their parameter choices in conditional design is a tremendous value of Scaffold-Lab.  Although additional studies addressing this are not required, the authors should at least discuss this point in a bit more detail.

**Author Replies:** Thank you for this valuable extended suggestion. We did want to explore different schemes of conditional design initially. However, it turned out that most of the conditional schemes are only achieved by certain methods but not popular enough to be benchmarked against each other; for example, the secondary structure conditioning were mainly achieved by RFdiffusion and Chroma, and domain classification conditioning was only enabled by Chroma, making it difficult for us to make a unified comparison among a series of different methods. Thus, we chose motif-scaffolding as the representative of conditional generation for the reason of (1) It is one of the most popular conditioning scheme achieved by different methods, making a comparison between different methods touchable; (2) It has numerous direct applications in the protein design problem, including but not limited to enzyme design, interface-conditioned backbone redesign, and vaccines. Thanks for your advice in additional assessments of specific cases of conditional design. In response, we decided to explore the active site-conditioned design as one of the most challenging scenarios of the motif-scaffolding problem.

We selected the catalytic sites of an artificial retro-aldolase, RA95.5-8F (PDB code: 5AN7), for assessing the four methods tested in Scaffold-Lab in terms of motif-scaffolding. The retro-aldolase catalyses the retro-aldol cleavage of R-methodol to 6-methoxy-2-naphthaldehyde and acetone. The RA95.5-8F was initially described in [3] and later redesigned using a specialized motif-scaffolding pipeline based on RFdiffusion, Riff-Diff [4]. The catalytic site consists of four residues which are spatially close to each other but sequentially discontinuous, which is typical geometric placement for enzyme active sites, making it an ideal case for investigating the active site-conditioned design problem. The successes by previous specialized methods also make us question whether modern motif-scaffolding methods could design it in a “zero-shot” or “one-shot” manner without additional information and expert knowledge of the active site.

We used the four tested methods, RFdiffusion, Chroma, GPDL and TDS, to design the retro-aldolase scaffold with 200 residues in total. Specifically, for RFdiffusion we tested two versions: (1) The base “motif-scaffolding” task which we have tested with the 24 cases in the previous version, and (2) The “enzyme design” task with an active site checkpoint (“<https://github.com/RosettaCommons/RFdiffusion?tab=readme-ov-file#the-active-site-model-holds-very-small-motifs-in-place>) which was fine-tuned with the task of scaffolding minimalist sites, along with implicit modeling of the substrate with an external potential described here (<https://github.com/RosettaCommons/RFdiffusion/blob/main/examples/design_enzyme.sh>). We termed the latter one “**RFdiffusion-enzyme**”. We intend to use this experiment to see how significant the active-site fine-tuning and substrate potential could help improve the success rates of active-site conditioned generation.

For the other methods, we solely used the four active site residues as input to the model. 100 scaffolds were generated by each method and evaluated with the Scaffold-Lab pipeline. We examine them with the criteria of success the same as described in the main text, and the results are provided in Table 1 below.

| Method | Success Rate (%) |
| --- | --- |
| RFdiffusion | 0 |
| Chroma | 0 |
| TDS | 0 |
| GPDL | 0 |
| RFdiffusion-enzyme | 20 |

**Table 1. Performances of motif-scaffolding methods in scaffolding the active sites of the retro-aldolase RA95.5-8F.**

We found the base versions for all four methods failed to produce a single solution for the active site of retro-aldolase, highlighting the difficulty of scaffolding the motifs containing multiple discontinuous residues. We hypothesize this could be due to the fact that the information contained in the input catalytic residues is too less as the constraint for the task of conditional generation. Surprisingly, we found RFdiffusion-enzyme succeeded 20 out of 100, which is a considerable success rate for such a challenging task. This highlights the importance for the minimalist site fine-tuning and the external information from the substrate. We suggest that these strategies could be insightful ones for method developments of the motif-scaffolding task in the future, particularly for methods tailored for minimalist sites motif-scaffolding, such as the enzyme design task.

Finally, we will add this part of extended experiments as an additional table in the Supplementary Material, along with the following paragraph in **Page 21-22**:

*“Motif-scaffolding has numerous applications in protein engineering, with one being the catalytic site conditioned motif-scaffolding which aims to design a novel enzyme scaffold starting with a set of catalytic motif residues. Therefore, we tested the motif-scaffolding methods with active sites of an enzyme, RA95.5-8F, which is an artificial retro-aldolase catalyzing a retro-aldol cleavage reaction. This enzyme was originally designed through directed evolution [3] and later redesigned using a well-established motif-scaffolding pipeline based on RFdiffusion, Riff-Diff [4], with a considerable success rate. Here we select this enzyme to see whether current motif-scaffolding methods are capable of scaffolding such enzyme catalytic sites starting with minimal inputs. Specifically, aside from the four tested methods described above, we additionally use an active site-specialized version RFdiffusion which was fine-tuned with minimalist sites along with implicit modeling of the substrate with an external potential, which we termed “RFdiffusion-enzyme”. We intended to explore whether this strategy could shed light on the catalytic enzyme scaffolding task.*

*The success rates of five tested methods are displayed in* ***Table 1****. We found the base versions for all four methods failed to produce a single solution for the active site of retro-aldolase, highlighting the difficulty of scaffolding the motifs containing multiple discontinuous residues. We hypothesize this could be due to the fact that the information contained in the input catalytic residues is too less as the constraint for the task of conditional generation. Surprisingly, we found RFdiffusion-enzyme succeeded 20 out of 100, which is a considerable success rate for such a challenging task. This highlights the importance for the minimalist site fine-tuning and the external information from the substrate. We suggest that these could be insightful strategies for method developments of the conditional generation task in the future, particularly for methods tailored for minimalist sites motif-scaffolding, such as the enzyme design task.”*

8. Although a web server is not provided, there appears to be an extensive github site which will allow scientists to use Scaffold-Lab in their research

**Author Replies:** We appreciate your positive comment on our GitHub repository. While a web server would definitely bring convenience for users, the complete evaluation of a single protein backbone generative model typically requires tens to hundreds of GPU hours, making it challenging to maintain such computational resources. However, to further aid the usability and make Scaffold-Lab more user-friendly, we have provided a Colab Notebook (<https://colab.research.google.com/github/Immortals-33/Scaffold-Lab/blob/main/scaffold_lab.ipynb>) which enables the evaluation of a couple of scaffolds for both unconditional generation and motif-scaffolding, along with more readable outputs such as a summary output file and PyMol session files containing successful scaffolds. This has been tested and we expect it to serve as an accessible entrance for users less familiar with the evaluation task as well as Scaffold-Lab.

Minor points

page 3. Cartesian should be capitalized

**Author Replies:** We have capitalized it now.

Supplementary Materials should be capitalized throughout

**Author Replies:** We have also capitalized them.

 Fig.3  Define "coll percentage" on Y axis

**Author Replies:** We have added a sentence to the legend of Figure 3 describing the definitions of helix and coils: *“The SSEs per residue are based on the assignment from DSSP, where α-helices are defined as the residues annotated with “H”, “G” or “I”; “coil are defined as the residues annotated as “C” or “S”.”* A more detailed explanations of the SSEs calculation could be found in the “Structural Properties” section in Supplementary Information.

At several points, the English needs some refinement. For example:

page 17. "Recently studies leveraging diffusion models to generate intrinsically disordered regions of proteins have shown great potentials applicated in both protein..."

**Author Replies:** We would like to thank the reviewer for the suggestion. We have rephrased it now:

“Diffusion models have lately been applied to model intrinsically disordered regions in both protein design and protein ensemble generation.”

Fig 3 legend. "All data was curated by directly calculating onto protein backbones without refolding"

**Author Replies:** We have replaced the sentence with the following one: “The analysis was performed onto the original backbone generated from each method”, for which we hope the point is made clearer now.

**References**

1. C. J. Williams et al. **MolProbity: More and better reference data for improved all-atom structure validation.** 2018, Protein Science
2. E. Papaleo et al. **The role of protein loops and linkers in conformational dynamics and allostery.** 2016, Chemical Reviews.
3. R. Obexer et al. **Emergence of a catalytic tetrad during evolution of a highly active artificial aldolase.** 2017, Nature Chemistry.
4. M. Braun et al. **Computational enzyme design by catalytic motif scaffolding.** 2025, Nature.
